# Uncovering Functional Genetic Variation in the Indigenous Greek Eghoria Goat: An Integrative Genome-Wide Discovery and Validation Approach

**DOI:** 10.3390/cimb48080778

**Published:** 2026-07-30

**Authors:** Maria-Anna Kyrgiafini, Georgios Stamatellos, Costas Stamatis, Zissis Mamuris

**Affiliations:** Laboratory of Genetics, Comparative and Evolutionary Biology, Department of Biochemistry and Biotechnology, University of Thessaly, 41500 Larissa, Greece; mkyrgiafini@uth.gr (M.-A.K.); stamatellosgeo@gmail.com (G.S.); kstamatis@bio.uth.gr (C.S.)

**Keywords:** *Capra hircus*, Eghoria goat, livestock, WGS, genome sequencing, genomic variation, MassARRAY, indigenous breed

## Abstract

The Eghoria goat constitutes the major indigenous goat population in Greece and represents an important genetic resource. Despite its significance, genomic information remains scarce. This study aimed to investigate coding variation in the Eghoria goat using whole-genome sequencing (WGS), with particular focus on missense single nucleotide polymorphisms (SNPs) and coding insertions/deletions (indels). Whole-genome sequencing was performed on Eghoria goats (*n* = 6), followed by bioinformatic processing and variant filtering. Functional characterization was conducted through Gene Ontology (GO) and KEGG pathway analyses (FDR < 0.05). Normalized variant density metrics identified highly polymorphic genes. Selected missense SNPs were validated in an independent population (*n* = 54) using MassARRAY genotyping. Whole-genome analysis identified 10,796,211 SNPs and 1,022,779 indels. After prioritization, 15,949 missense SNPs and 861 indels were retained. Functional enrichment analyses highlighted pathways related to metabolism, ion transport, calcium signaling, immune function, transcriptional regulation, and environmental adaptation. Genes exhibiting elevated polymorphism density included olfactory receptor family members, and loci associated with metabolic regulation. Validation analyses confirmed the presence of selected variants in the Eghoria goat population. The study expands current knowledge of genomic diversity in the indigenous Eghoria goat and provides valuable resources for future genetic improvement, conservation, and breeding programs.

## 1. Introduction

Livestock production is a cornerstone of global agricultural systems, contributing substantially to food security, rural development, and economic sustainability [1]. Among livestock species, goats (*Capra hircus*) occupy a particularly important position because of their remarkable adaptability to diverse and often harsh environmental conditions, as well as their contribution to milk and meat production [2,3]. In Greece, goat farming represents a major agricultural activity with considerable socioeconomic and environmental importance [4]. The country maintains the largest goat population in the European Union (EU), comprising approximately 2.58 million animals in 2024, and is among the leading goat milk producers in the EU [5]. Beyond their productive role, goats in Greece are closely associated with traditional farming practices and local ecosystems, with indigenous breeds serving as a valuable reservoir of genetic diversity and adaptation.

Indigenous goat populations are characterized by their resilience to environmental stress, ability to utilize low-quality feed resources, and resistance to local diseases [6]. These traits make them particularly valuable in the context of climate change and the need for sustainable livestock production systems [7]. However, numerous indigenous Greek goat populations are currently facing demographic decline and genetic erosion, primarily attributable to production intensification and indiscriminate crossbreeding practices. Specifically, high-yielding cosmopolitan breeds, such as Saanen, Alpine, Toggenburg, and Anglo-Nubian, which have been widely introduced into Greece [4], are often used in uncontrolled crossbreeding with indigenous goat populations. This practice can lead to a reduction in the number of purebred native animals, loss of genetic diversity, and the erosion of unique traits associated with economic value and local adaptation. Given the increasing vulnerability of the Mediterranean region to climate change, characterized by rising summer temperatures and prolonged drought periods, the conservation and study of locally adapted goat populations are becoming increasingly critical [8]. The protection of these genetic resources is essential not only for the preservation of biodiversity but also for ensuring the long-term sustainability and resilience of livestock production systems in Greece.

Greek goat populations are predominantly represented by two main indigenous breeds, Eghoria and Skopelos, with the former accounting for approximately 90% of the national herd and exhibiting a wide geographical distribution across the country. These breeds are primarily reared for milk production, which supports the manufacture of numerous traditional dairy products, many of which are recognized as Protected Designation of Origin (PDO) or Protected Geographical Indication (PGI). The Eghoria goat is characterized by pronounced phenotypic diversity, including variation in coat color (black, brown, white, or mixed patterns) and long hair, reflecting its adaptability to a wide range of environmental conditions. Most animals are reared under low-input, semi-extensive farming systems, typically in mountainous or semi-mountainous areas characterized by heterogeneous and often challenging environmental conditions. It is a dual purpose breed that typically produces 100–250 kg of milk per lactation, while maintaining the ability to efficiently utilize low-quality pastures and tolerate hot and dry climatic conditions [4,9]. Thus, the Eghoria breed represents a particularly valuable genetic resource due to its combination of phenotypic variability, environmental adaptability, and production potential. These characteristics highlight its importance for sustainable livestock production and underline the need for its comprehensive genomic characterization and conservation.

Genomic studies of Greek goat populations remain relatively limited, particularly concerning the Eghoria breed, which has received comparatively little attention [10]. Most available studies have focused on a small number of genetic loci or on associations with specific traits [11,12,13], while population-level analyses are scarce and often rely on SNP microarrays [14,15]. The introduction of high-density SNP arrays marks a substantial methodological advancement, enabling large-scale analyses of genetic diversity, inbreeding, and selection signatures. However, these approaches are limited in their capacity to represent the full spectrum of genomic variation, as they are constrained by predefined marker sets, thereby restricting their ability to comprehensively capture genome-wide variation.

Recent advances in next-generation sequencing (NGS) technologies have significantly transformed the field of livestock genomics by enabling high-resolution analyses of genetic variation across entire genomes [16,17,18]. Specifically, whole-genome sequencing provides a robust framework for the comprehensive identification of single nucleotide polymorphisms (SNPs) and insertions/deletions (indels), thereby facilitating the exploration of functionally relevant genetic variation. As sequencing costs continue to decrease, WGS is becoming an increasingly accessible and informative tool for livestock genomic studies [17,19]. In the context of Greek goat populations, a recent study generated WGS data from a limited cohort of indigenous Greek goats (*n* = 14), providing initial insights into genomic variation. However, this study was constrained by low sequencing depth (~3.18× coverage) and primarily focused on data generation and variant detection, without the integration of systematic functional prioritization or experimental validation approaches [20]. Consequently, high-resolution, genome-wide studies focusing specifically on the Eghoria goat remain scarce, particularly those integrating variant discovery, functional interpretation, and validation at the individual level. This gap limits our understanding of the genetic underpinnings of adaptation and constrains the application of genomic information in conservation efforts, traceability, and precision breeding strategies. Addressing these limitations is particularly critical in the context of climate change and the ongoing transformations within agricultural systems, which exert additional pressures on locally adapted genetic resources.

In this context, the aim of the present study was to characterize coding sequence variation in the Eghoria goat, which serves as a representative example of a locally adapted and underexplored genetic resource, utilizing a whole-genome sequencing approach. Emphasis was placed on the identification and filtering of functionally relevant missense single nucleotide polymorphisms (SNPs) and exonic insertions/deletions (indels), given their potential impact on protein function and their contribution to adaptive traits. To further elucidate the biological significance of the identified variants, functional annotation and enrichment analyses were performed using Gene Ontology (GO) and Kyoto Encyclopedia of Genes and Genomes (KEGG) frameworks. Furthermore, a subset of candidate variants was validated at the individual level using MassARRAY genotyping to confirm their presence and assess their distribution within the population. Through this integrative approach, which combines genome-wide variant discovery, functional interpretation, and experimental validation, the current study provides novel insights into the genetic architecture of the Eghoria goat and contributes to ongoing efforts toward the conservation and sustainable management of indigenous livestock resources.

## 2. Materials and Methods

### 2.1. Biological Material and DNA Extraction

**Figure 1:**
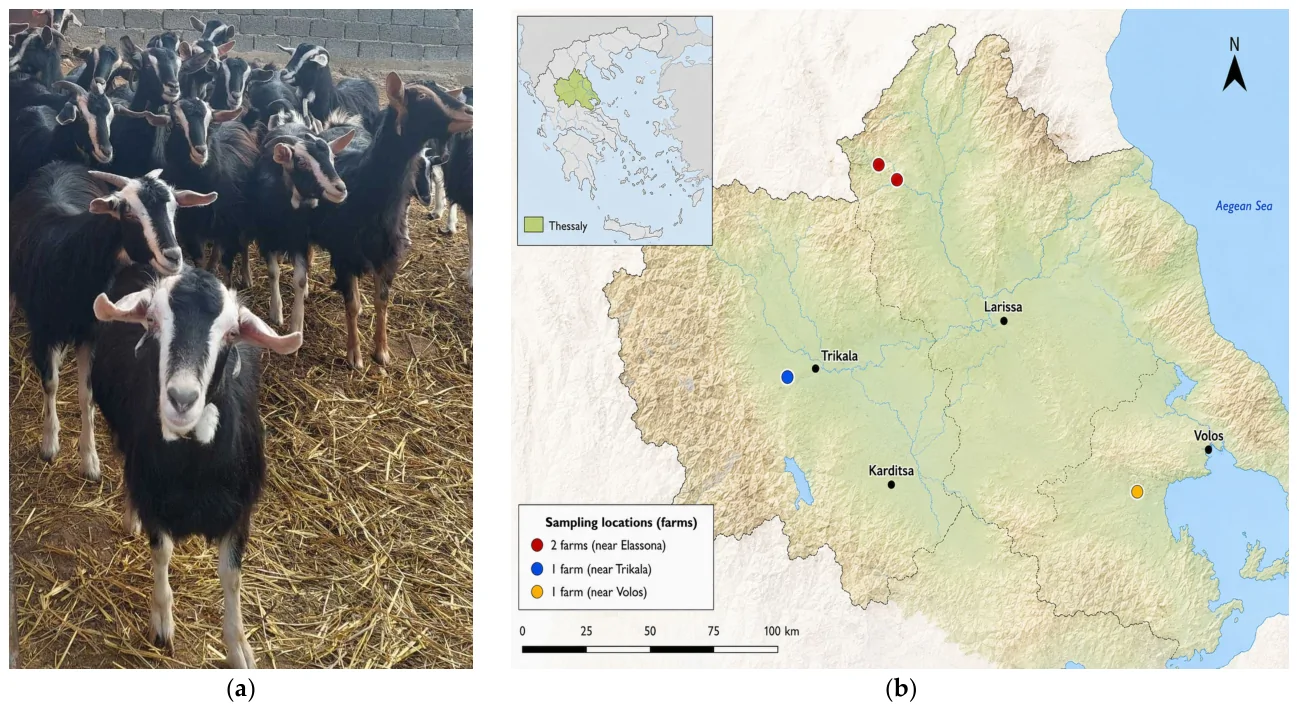
Representative animals of the Greek Eghoria goat breed sampled in this study (**a**); Map showing the geographical distribution of the Eghoria goat farms included in this study within the Thessaly region, Greece (**b**).

Blood samples were collected from a total of 60 (*n* = 60) Eghoria goats (*Capra hircus*), an indigenous breed of Greece, from farms located in Thessaly region (Figure 1). All animals were sourced from commercial farming systems, and the selection process aimed at minimizing relatedness based on available farm records. The sampled population consisted of clinically healthy animals of productive age, with approximately equal numbers of males and females. All procedures involving animals were conducted in accordance with European animal welfare regulations and the study protocol received approval by the Ethics Committee and Research Ethics Board (E.H.D.E.) of the University of Thessaly (Approval Code: 27/07-04-2023). Blood collection was performed by trained personnel following standardized practices to ensure minimal stress on the animals.

The study population was structured according to the experimental design: six individuals (*n* = 6) were selected for the whole-genome sequencing phase, while the remaining fifty-four (*n* = 54) animals comprised an independent cohort utilized for validation through MassARRAY genotyping.

Whole blood samples were collected in EDTA tubes, transported under cooled conditions, and stored at −20 °C until further processing. Genomic DNA was extracted using the PureLink Genomic DNA Mini Kit (Thermo Fisher Scientific, Waltham, MA, USA), following the manufacturer’s instructions. DNA concentration was quantified using a Qubit 4 Fluorometer in combination with the Qubit dsDNA BR Assay Kit (Thermo Fisher Scientific, Waltham, MA, USA), while DNA quality and integrity were assessed through agarose gel electrophoresis. Only samples that met the predefined quality criteria were retained for subsequent WGS and genotyping analyses.

### 2.2. Library Preparation and Whole-Genome Sequencing

Genomic DNA from six selected individuals was pooled in equimolar concentrations to generate a representative sample of the population. The sample was sent to Novogene Co., Ltd. (Munich, Geermany) for library preparation and WGS.

Library preparation was conducted utilizing a standard Illumina-compatible workflow. Briefly, genomic DNA was fragmented into short segments, followed by end-repair and A-tailing, with subsequent ligation of full-length sequencing adapters. Size selection was performed to obtain libraries with an average insert size of approximately 350 bp. Polymerase chain reaction (PCR) amplification was performed as necessary, and the final libraries were purified using the AMPure XP system (Beckman Coulter, Brea, CA, USA). Library quality and fragment size distribution were assessed using the Agilent Fragment Analyzer System, while library concentration was determined using both the Qubit Fluorometer (Thermo Fisher Scientific, Waltham, MA, USA) and quantitative PCR (qPCR), followed by normalization prior to sequencing.

Sequencing was performed using the Illumina NovaSeq X Plus platform, generating paired-end reads of 150 bp (PE150). The sequencing output yielded approximately 60 Gb of raw data, corresponding to an estimated coverage of ~22×. Raw sequencing data were provided in FASTQ format and used for downstream bioinformatic analyses.

### 2.3. Bioinformatics Analysis

Raw FASTQ files were initially subjected to quality assessment using FastQC [21] to evaluate read quality metrics and detect potential technical artifacts. Adapter sequences and low-quality bases (PHRED < 30) were trimmed using Trimmomatic (v0.39) [22]. The filtered reads were aligned to the *Capra hircus* reference genome (ARS1 assembly, Ensembl release 92) using the Burrows–Wheeler Aligner (BWA) (version 0.7.17) [23]. The resulting alignment files were processed using Picard tools (http://broadinstitute.github.io/picard/, accessed on 15 June 2026), where potential PCR duplicates were marked and removed to reduce biases in downstream analyses. SAM files were converted to sorted BAM files with SAMtools (v1.19.2) [24]. Variant discovery, including both single nucleotide polymorphisms (SNPs) and insertions/deletions (indels), was performed using the SAMtools mpileup/bcftools pipeline [24]. Raw variant calls were subsequently filtered using the same filtering criteria adopted in our previous study [25] to ensure methodological consistency. Candidate variants satisfying the predefined quality criteria (DP > 4 and MQ > 20) were retained for downstream annotation and prioritization. Genotype quality (GQ) filtering was not applied because individual genotypes cannot be reliably inferred from pooled sequencing data. Instead, prioritized candidate variants were subsequently validated by MassARRAY genotyping in an independent validation cohort. The resulting variant call format (VCF) files containing the final set of filtered SNPs and indels were used for downstream analyses. Functional annotation of the filtered variants was performed using ANNOVAR [26], which classified variants according to their genomic location (e.g., exonic, intronic, intergenic) and predicted their potential functional effects on protein-coding sequences. Annotation was based on available gene models and functional databases, enabling biological interpretation of the identified variants.

### 2.4. Variant and Indel Filtering, Annotation and Functional Enrichment Analysis

The primary objective of this analysis was to characterize and functionally interpret the genetic alterations identified in the Greek Eghoria goat, with a particular emphasis on those that may contribute to adaptive or biologically relevant traits. In this context, we prioritized missense SNPs and exonic insertions/deletions (indels), as these are more likely to affect protein structure and function, potentially resulting in phenotypic variation. The potential biological implications of the filtered variants were assessed through GO and KEGG pathway analyses. Furthermore, genomic regions exhibiting high SNP or indel density were identified, as these regions may represent loci associated with critical physiological or adaptive processes.

Initially, two annotated variant datasets were constructed: one consisting of exonic SNPs and the other of exonic indels. Each dataset included essential variant information, such as chromosome (CHR), genomic position (POS), reference (REF) and alternate (ALT) alleles, gene annotation, and variant identifiers (rsID), when available. Preliminary quality control procedures involved the removal of duplicate entries and variants lacking or containing invalid gene annotations. For SNPs, only missense variants were retained, whereas all exonic indels were included due to their potential implications for coding regions.

Genes harboring missense SNPs and exonic indels were extracted and analyzed independently, yielding two distinct gene sets. These gene lists were subjected to exploratory functional enrichment analysis utilizing ShinyGO v. 0.82 [27], with *Capra hircus* specified as the reference organism. Enrichment analyses were performed using the default statistical settings of the platform, with multiple testing correction applied using the false discovery rate (FDR) method. A significance threshold of adjusted *p* < 0.05 was used to identify enriched terms. Enrichment analyses included GO categories (Biological Process, Molecular Function, Cellular Component) [28,29] as well as KEGG pathways [30], enabling the identification of overrepresented biological functions and pathways.

To conduct a more comprehensive analysis of the genomic distribution of variants, variant counts were computed separately for SNPs and indels, yielding metrics termed “SNP Count per Gene” and “Indel Count per Gene.” Gene coordinates were obtained from Ensembl BioMart [31] to determine gene lengths in kilobases (kb). Subsequently, variant densities were normalized to express SNPs per kilobase (SNPs/kb) and indels per kilobase (indels/kb). These metrics facilitated the identification of genes with heightened polymorphism densities, which may suggest regions of potential functional significance or indicate putative selection signals.

All analyses were conducted using custom Python scripts in Python 3.12, primarily using the Pandas library (v2.2.2) [32].

### 2.5. Selection of Candidate Missense Variants for Validation

To validate and further investigate the most biologically relevant genetic variants identified through whole-genome sequencing, a subset of candidate missense SNPs was selected for downstream genotyping utilizing the MassARRAY platform.

Variant selection was conducted based on a comprehensive assessment of functional, genomic, and technical criteria. Specifically, priority was given to: (i) missense variants due to their potential impact on protein structure and function; (ii) variants located within genes identified as functionally relevant through GO and KEGG enrichment analyses; (iii) variants within genes exhibiting elevated normalized SNP densities (SNPs/kb), indicative of increased levels of polymorphism; and (iv) variants distributed across different genomic regions to ensure broad genome representation and to avoid clustering bias. In addition, variant-level quality metrics, including read depth, mapping quality, and overall variant confidence, were meticulously considered to ensure the selection of high-quality candidates. Variants were additionally screened for technical suitability for MassARRAY assay design, which included the exclusion of nearby polymorphisms that could potentially interfere with primer binding and compromise genotyping accuracy.

Based on these criteria, a final panel of 24 missense SNPs was selected for validation. The selected variants were subsequently genotyped in an independent cohort of 54 Eghoria goats utilizing the MassARRAY system. To further evaluate the potential biological relevance of the validated missense variants, functional impact predictions were obtained using SIFT [33], while evolutionary conservation was assessed using GERP (Genomic Evolutionary Rate Profiling) scores [34], where available.

### 2.6. MassARRAY Assay Design and Genotyping

Genotyping of the 24 selected missense SNPs was conducted utilizing the MassARRAY^®^ system (Agena Bioscience, San Diego, CA, USA), which employs matrix-assisted laser desorption/ionization time-of-flight (MALDI-TOF) mass spectrometry.

MassARRAY assays were designed and conducted by Inqaba Biotec™ (Inqaba Biotechnical Industries, Pretoria, South Africa), adhering to the established iPLEX genotyping protocol. In summary, genomic regions flanking each selected SNP were utilized to design locus-specific primers, enabling multiplex PCR amplification. During the assay design phase, the sequence context was meticulously assessed to minimize potential interference from adjacent polymorphisms and to ensure efficient amplification and extension. The genotyping workflow included multiplex PCR amplification of the target regions, followed by enzymatic cleanup employing shrimp alkaline phosphatase (SAP). Subsequently, a single-base extension reaction (iPLEX) was conducted utilizing mass-modified terminator nucleotides. The extension products were then deposited onto a SpectroCHIP array and analyzed via MALDI-TOF mass spectrometry, enabling allele discrimination based on mass differences [35].

Raw spectral data were processed utilizing MassARRAY Typer software 4.1 (Agena Bioscience), wherein genotype calls were assigned through automated clustering algorithms. Subsequently, genotype calls were evaluated for quality and consistency prior to conducting downstream statistical analyses.

### 2.7. Genotyping Quality Control and Statistical Analyses

Genotype data generated from the MassARRAY platform underwent rigorous quality control procedures. Both SNP-level and sample-level call rates were assessed to ensure data reliability. Variants and samples exhibiting low call rates, defined by a threshold set of ≥90% completeness, were excluded from further analysis. Additionally, genotype clustering quality was visually examined utilizing the MassARRAY Typer output to confirm clear allele discrimination and the consistency of genotype calls. Only high-confidence genotypes were retained for subsequent analyses.

Allele and genotype frequencies were calculated for all validated SNPs within the studied population. Descriptive statistics were used to summarize allele distributions and minor allele frequencies (MAF) across the dataset. MAF values were calculated to assess the level of polymorphism for each SNP. These analyses enabled the characterization of allele variation at the individual-animal level, following variant identification through the pooled WGS approach. Expected heterozygosity (He), observed heterozygosity (Ho), and polymorphism information content (PIC) were calculated for each validated biallelic SNP. Expected heterozygosity was calculated as He = 2pq, whereas observed heterozygosity (Ho) was calculated as the proportion of heterozygous individuals at each locus. Polymorphism information content was calculated as PIC = 1 − p^2^ − q^2^ − 2p^2^q^2^, where p and q represent the frequencies of the two alleles. Deviations from Hardy–Weinberg equilibrium (HWE) were evaluated for each locus using the observed genotype counts, with statistical significance set at *p* < 0.05.

All statistical analyses were conducted using R version 4.3.2. [36], employing standard packages for data management and summary statistics.

## 3. Results

### 3.1. Whole-Genome Sequencing and Data Quality

Whole-genome sequencing of the pooled Eghoria goat sample yielded approximately 60.8 Gb of raw data, corresponding to 400,183,414 paired-end reads. Following quality filtering, 60.0 Gb of clean data were retained, indicating a negligible data loss during preprocessing. The sequencing output corresponded to an estimated ~22× coverage, providing sufficient depth for reliable variant detection. Quality assessment demonstrated high sequencing accuracy, with Q20 values exceeding 97% and Q30 values above 93% across all lanes, indicating that the majority of bases were of high quality. The overall sequencing error rate was low (~0.01%), and GC content was consistent (~44%), suggesting the absence of significant technical biases.

High-quality reads were aligned to the *Capra hircus* reference genome (ARS1 assembly), resulting in 398,298,031 mapped reads out of a total of 400,183,414 reads, corresponding to a mapping rate of 99.53%.

### 3.2. Variant Identification, Prioritization and Genomic Distribution

Whole-genome variant analysis identified a total of 10,796,211 single nucleotide polymorphisms (SNPs) and 1,022,779 insertions/deletions (indels). SNPs accounted for the majority of the detected variants, consistent with typical patterns observed in mammalian genomes. Functional annotation revealed that most variants were located in intergenic and intronic regions, with a smaller proportion mapped to coding sequences. Specifically, 2,919,335 SNPs (28%) were found in intronic regions, while 7,155,609 SNPs (69%) were located in intergenic regions. Similarly, 281,637 indels (29%) were classified as intronic, and 675,634 indels (69%) were categorized as intergenic.

To further investigate coding sequence variation in the Eghoria goat (*Capra hircus*), variant prioritization was conducted with a focus on exonic missense SNPs and exonic indels, due to their higher likelihood of affecting protein function and contributing to phenotypic variability. After implementing filtering and prioritization criteria, a total of 15,949 missense SNPs and 861 exonic indels were retained for subsequent analyses.

**Figure 2:**
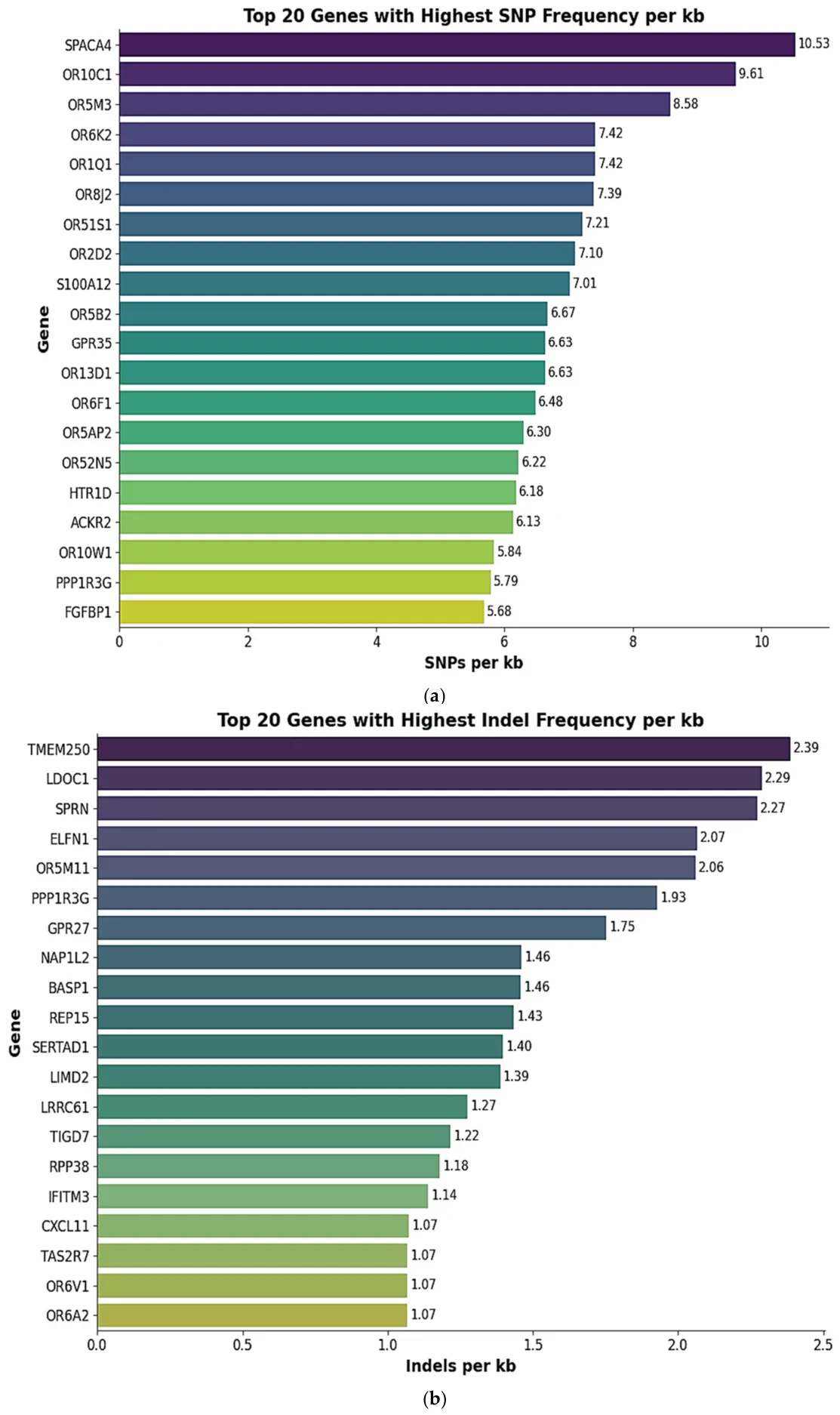
Genes with the highest normalized variant density in the Greek Eghoria goat are presented. Normalized variant densities, expressed as single nucleotide polymorphisms per kilobase (SNPs/kb) and insertions/deletions per kilobase (indels/kb), were calculated for all genes to account for differences in gene length. Figures show the genes with the highest SNP density (**a**) and the highest indel density (**b**). Only the top 20 genes per category are displayed.

To evaluate the genomic distribution of these variants, SNPs and indels were mapped to their corresponding protein-coding genes. Variant density was normalized by gene length and expressed as the number of variants per kilobase (SNPs/kb and indels/kb), allowing comparisons across genes of different sizes. This approach enabled the identification of genes with elevated levels of coding sequence polymorphism. Genes with the highest normalized SNP densities included *SPACA4*, *OR10C1*, *OR5M3*, *OR1Q1*, and *OR6K2* (Figure 2a), while those with the highest indel densities included *TMEM250*, *LDOC1*, *SPRN*, *ELFN1*, and *OR5M11* (Figure 2b). The exact normalized SNP and indel density values for all analyzed genes are provided in Table S1. Notably, a substantial proportion of the top-ranking genes for SNP density belonged to the olfactory receptor (OR) gene family, which is known for its high variability and rapid evolution in mammals [37]. Conversely, genes characterized by high indel densities exhibited a more functionally diverse profile, including genes implicated in regulatory, neuronal, and immune-related processes. Overall, genes with elevated variant densities may indicate genomic regions of increased variability and potential functional significance within the Eghoria goat genome.

### 3.3. Functional Enrichment Analysis

To elucidate the biological significance of genes harboring prioritized coding variants, a functional enrichment analysis was conducted utilizing ShinyGO v0.82 [27]. The analyses were performed separately for genes with prioritized missense SNPs and exonic indels, thereby facilitating the identification of potentially distinct functional patterns associated with each variant type. Enrichment results were evaluated across GO categories, Biological Process (BP), Molecular Function (MF), and Cellular Component (CC), as well as KEGG pathways, to discern significantly overrepresented biological functions and pathways. This methodological approach enabled the identification of biological processes and pathways potentially associated with coding sequence variation in the Eghoria goat genome.

#### 3.3.1. Missense SNP Enrichment Analysis

Functional enrichment analysis of genes harboring missense SNPs revealed several biologically coherent functional clusters. At the Biological Process (BP) level, the enriched terms were predominantly associated with responses to stimuli and regulatory processes, alongside developmental and differentiation pathways, including multicellular organism development and anatomical structure formation. A secondary cluster was related to cellular organization and morphogenesis, suggesting potential roles in tissue development and structural organization. At the Molecular Function (MF) level, the enrichment analysis revealed a predominance of ion channel and transmembrane transporter activities, with a particular emphasis on cation transport and passive membrane transport. Additional terms associated with catalytic and phosphotransferase activities were also enriched, suggesting involvement in signaling cascades and protein modification processes. In the Cellular Component (CC) category, the enriched terms were predominantly associated with membrane-related regions and extracellular structures, including the plasma membrane, cell periphery, and extracellular matrix. Enrichment was also observed in intracellular compartments, such as nuclear and organelle lumen structures, suggesting a broad subcellular distribution of the affected gene products.

Consistent with these findings, KEGG pathway analysis revealed significant enrichment in pathways related to cell–environment interactions and signaling mechanisms, including ECM–receptor interactions and the calcium signaling pathway. Additional enriched pathways included transport and sensory-related processes, such as ABC transporters and taste transduction, as well as neuroactive signaling pathways. Broader pathways associated with metabolism were also identified, reflecting the involvement of various biochemical processes. Furthermore, enrichment in pathways linked to the innate immune system, including complement and coagulation cascades, was observed.

Overall, these findings indicate that missense SNPs are preferentially situated within genes associated with signal transduction and membrane-related processes, particularly those pertaining to ion transport, cellular communication, and developmental regulation. The enrichment of pathways linked to cell–environment interactions, sensory perception, and immune-related processes further emphasizes the potential functional relevance of these variants within the Eghoria goat genome. The most significantly enriched GO terms and KEGG pathways are summarized in Table 1. The complete GO and KEGG enrichment results, including Fold Enrichment, enrichment FDR (adjusted *p* values), and the number of genes associated with each enriched term, are provided in Supplementary Tables S2–S5.

#### 3.3.2. Exonic Indels Enrichment Analysis

Functional enrichment analysis of genes harboring exonic indels demonstrated distinct biological patterns in comparison to missense SNPs. At the Biological Process (BP) level, the enriched terms were predominantly associated with developmental and system-level processes, including multicellular organism development, circulatory system development, and anatomical structure formation. In addition, terms related to negative regulation of cellular and biological processes, as well as cellular component organization, were significantly enriched, indicating potential roles in regulatory and structural cellular functions. At the Molecular Function (MF) level, enrichment was largely driven by activities related to the regulation of signaling and gene expression, including calcium-dependent phosphatase activity, GTPase regulator activity, and transcription factor binding. Notably, several enriched terms were associated with DNA binding and transcriptional regulation, suggesting that indels may preferentially impact genes implicated in the regulation of gene expression. In the Cellular Component (CC) category, the enriched terms were predominantly linked to nuclear and intracellular compartments, including the nucleoplasm, nuclear lumen, and organelle lumen, as well as membrane-associated structures such as the plasma membrane and cell periphery. The inclusion of terms such as adherens junction further indicates a potential involvement in cell–cell interactions and tissue organization. No significantly enriched KEGG pathways were identified for indel-associated genes.

Overall, these results suggest that exonic indels are enriched in genes involved in developmental processes, transcriptional regulation, and intracellular organization, highlighting their potential functional impact on gene regulation and cellular architecture within the Eghoria goat genome. The most significantly enriched GO terms are summarized in Table 2. The complete GO enrichment results, including Fold Enrichment, enrichment FDR (adjusted *p* values), and the number of genes associated with each enriched term, are provided in Supplementary Tables S6–S8.

### 3.4. Validation of Candidate Missense SNPs Using MassARRAY Genotyping

To validate candidate variants identified through the pooled WGS approach at the individual-animal level, a subset of 24 candidate missense SNPs was selected for genotyping utilizing the MassARRAY platform in an independent cohort of 54 Eghoria goats. This validation step aimed to confirm that variants detected in the pooled sequencing dataset were reproducible and polymorphic in individual samples, thereby supporting the reliability of the variant discovery and prioritization methodology. The variants were selected based on criteria including their functional relevance, genomic distribution, and technical suitability for assay design, as delineated in the Section 2. The selected SNPs were distributed across genes involved in various biologically significant functions.

Genotyping was successfully conducted for the selected SNP panel, permitting subsequent evaluation of genotyping quality and allele frequency distribution within the population. Detailed information for all selected variants, including genomic position, rsID (when available), reference and alternate alleles, and gene symbol, is presented in Supplementary Table S9. To further characterize the validated missense variants, SIFT predictions and GERP conservation scores were also evaluated (Table S9). Most variants were predicted to be tolerated by SIFT, whereas three variants, located in *OR1Q1*, *OR6F1* and *GPR35*, were predicted to be deleterious. GERP scores were available for a subset of variants and generally indicated limited evolutionary conservation at the corresponding genomic positions.

#### 3.4.1. Genotyping Quality and Call Rates

Genotyping quality was evaluated at both the sample and SNP levels to ensure the reliability of the MassARRAY results.

At the sample level, all 54 individuals were successfully genotyped, with call rates ranging from 90% to 100%. The majority of samples achieved call rates of ≥93%, with several samples exhibiting complete genotype recovery (100%), thereby indicating an overall high level of genotyping performance across the cohort (Supplementary Table S10). At the SNP level, most variants demonstrated high coverage and robust amplification, with the majority of SNPs attaining call rates of ≥95%. A limited number of SNPs exhibited slightly reduced call rates (e.g., 90–92.5%); however, these still met the predefined quality threshold of ≥90% completeness and were retained for subsequent analyses (Supplementary Table S10). Visual inspection of genotype clustering confirmed clear and consistent allele separation, thereby supporting accurate genotype assignment.

Overall, the high call rates observed across both samples and SNPs indicate that the selected SNP panel and assay design were particularly effective and reliable for genotyping within the Eghoria goat population.

#### 3.4.2. Allele Frequency Distribution

Allele and genotype frequencies were computed for all validated SNPs within the Eghoria goat population. The analyzed variants exhibited a range of allele frequencies, indicating the presence of common polymorphisms across all examined loci.

The allele frequencies ranged from 0.54 to 0.88, with the majority of SNPs displaying moderate to high frequencies, suggesting that these variants are well represented within the population. No SNPs with extremely low allele frequencies were observed, indicating the absence of rare variants among the selected panel. Importantly, the observed allele frequency distribution confirms that the variants initially identified through the pooled WGS approach are also detectable at the individual level, thereby supporting their biological validity and excluding potential sequencing artifacts. The predominance of intermediate-to-high frequency variants further suggests that these loci represent established polymorphisms within the Eghoria goat population.

Expected heterozygosity (He) ranged from 0.211 to 0.497, observed heterozygosity (Ho) ranged from 0.184 to 0.482, and PIC values ranged from 0.189 to 0.373. Several loci exhibited He values above 0.40 and PIC values above 0.30, indicating appreciable genetic variability among the validated variants. The *ABCA5* variant showed the lowest He and PIC values. In addition, none of the validated loci deviated significantly from Hardy–Weinberg equilibrium (*p* > 0.05).

Detailed genotype validation statistics, including allele frequencies, He, Hο, PIC values, and HWE results are presented in Supplementary Table S11.

## 4. Discussion

Indigenous breeds represent a significant reservoir of genetic diversity, shaped by long-term adaptation to local environments, management practices, and production systems [6]. In the present study, we comprehensively characterized coding sequence variation in the Eghoria Greek goat employing a whole-genome sequencing approach, combined with functional annotation and experimental validation. Following the prioritization of missense SNPs and exonic indels, we identified variants with potential functional relevance distributed across the genome. Functional enrichment analyses revealed that these variants are associated with key biological processes, including signal transduction, developmental pathways, and transcriptional regulation. Notably, validation of selected SNPs using MassARRAY genotyping confirmed the presence and polymorphic nature of the identified variants, supporting the reliability of the variant discovery pipeline. In addition, the validated loci exhibited observed heterozygosity values consistent with the expected genetic variation and did not show significant deviations from Hardy–Weinberg equilibrium, further supporting the robustness of the genotyping results.

At first, the genome-wide distribution of variants identified in this study follows patterns commonly observed in mammalian genomes and specifically in goats [20,38,39]. The majority of SNPs and indels are located in intergenic and intronic regions, with a smaller proportion affecting coding sequences, reflecting the predominance of non-coding DNA within the goat genome. However, variants within coding regions, particularly missense SNPs and exonic indels, are of particular interest due to their potential to directly impact protein structure and function. Similar prioritization strategies that focus on potentially functional coding variants have been extensively utilized in livestock genomics to identify candidate loci associated with critical biological and productive traits [25,40,41,42].

Following bioinformatics filtering, functional enrichment analyses of genes harboring prioritized missense SNPs and exonic indels indicated significant involvement in biological pathways related to signal transduction, developmental regulation, cellular organization, and metabolic processes, highlighting the potential functional relevance of the identified coding variants in the Eghoria goat genome. Furthermore, while missense SNPs and exonic indels displayed partially overlapping enrichment profiles, distinct functional patterns were also identified between the two variant categories.

Genes containing missense SNPs were predominantly enriched in pathways associated with ion transport, transmembrane signaling, calcium signaling, extracellular matrix (ECM) interactions, and metabolism, suggesting their potential involvement in physiological adaptation of the Eghoria goat. Ion transport and calcium signaling are fundamental regulators of cellular communication, homeostasis, and stress responses and have previously been implicated in livestock adaptation to environmental challenges, including heat stress, high-altitude hypoxia [43,44,45] and nutritional constraints [46]. These findings align with previous research; a recent study identified Runs of Homozygosity (ROHs) containing genes associated with heat stress response and resilience in Greek goats, confirming their potential for adaptation to local semi-arid and hot-arid environments [15]. Moreover, the enrichment of ATP-binding cassette (ABC) transporter pathways may be related to molecular transport, detoxification processes, and cellular protection mechanisms [47], which are vital for adaptation to heterogeneous and low-input production systems. Similarly, the enrichment of metabolic pathways highlights the importance of efficient energy utilization and metabolic regulation in locally adapted goat populations. Enrichment of the ECM–receptor interaction pathway further suggests the involvement of tissue organization and muscle development, cellular communication, and physiological adaptation [48,49]. Other enriched pathways may also provide insights into the adaptive characteristics of the Eghoria goat. The complement and coagulation cascades pathway is associated with innate immunity, inflammatory responses, and tissue repair, suggesting a potential role in maintaining health and resilience under challenging environmental conditions. Lysine degradation is involved in amino acid and energy metabolism, which may contribute to metabolic flexibility and adaptation to variable nutritional resources. Similarly, the taste transduction pathway may also contribute to environmental adaptation by influencing feed preference, dietary selection, and the efficient utilization of diverse forage resources. These functions are particularly relevant for indigenous goat breeds, which often thrive under extensive grazing systems and exploit heterogeneous vegetation in challenging environments. Therefore, variation in genes associated with this pathway may reflect adaptive mechanisms that support nutritional flexibility and resilience.

In addition to the functional enrichment analyses, the evaluation of normalized SNP densities revealed genes with elevated levels of coding sequence polymorphism within the Eghoria goat genome, many of which belonged to the olfactory receptor (OR) gene family (*OR10C1*, *OR5M3*, *OR1Q1*, *OR6K2*, *OR51S1)*. OR genes represent one of the most diverse and rapidly evolving gene families in mammals and have been associated with sensory perception, feeding behavior, reproduction, and environmental adaptation in goats and other livestock species [50,51]. Consistent with our findings, previous genomic studies have reported enrichment of OR genes in genomic regions under selection in indigenous sheep and goat populations [25,52,53]. Therefore, the high variability observed may reflect adaptive processes that facilitate the efficient exploitation of heterogeneous environments and forage resources under the extensive production systems in which Eghoria goats are traditionally reared.

Beyond OR-related genes, elevated SNP densities were also observed in genes associated with immune response, metabolic regulation, and cellular signaling, suggesting that multiple biological systems may contribute to the adaptive genomic landscape of the Eghoria goat. Among these, *S100A12* is involved in innate immunity and inflammatory responses [54], whereas variation in *ACKR2* has previously been reported in the adaptable Pırlak sheep breed [42]. Similarly, *FGFBP1*, involved in fibroblast growth factor signaling, has been linked to metabolism, tissue remodeling, and muscle development in livestock [55], while *HTR1D* has been associated with mammary gland function during lactation in small ruminants [56]. Together, these findings identify additional candidate genes that may contribute to adaptation and other physiologically important traits in the Eghoria goat.

In contrast, genes harboring exonic indels were predominantly enriched in pathways related to development, transcriptional regulation, intracellular organization, and methyltransferase complexes, suggesting that these variants preferentially affect regulatory and organizational cellular functions. Enrichment of DNA-binding and transcriptional regulatory activities further supports their potential role in modulating gene expression and broader biological responses. Collectively, these findings indicate that missense SNPs and exonic indels contribute to distinct yet complementary aspects of genomic and functional diversity in the Eghoria goat.

Consistent with these enrichment patterns, genes harboring elevated indel densities included loci associated with cellular regulation, immune-related processes, signaling pathways, and metabolism, such as *PPP1R3G*, *IFITM3*, and *CXCL11*. The identification of immune-related genes, such as *IFITM3*, supports the potential contribution of structural variation to immune responsiveness and environmental resilience in locally adapted goat populations [57]. Increased indel density was also observed in *SPRN*, a gene previously associated with susceptibility to atypical scrapie in sheep [58], highlighting its potential biological relevance in small ruminants. In addition, *ELFN1* and *SERTAD1*, previously linked to growth-related traits and mastitis susceptibility, respectively, represent additional candidate genes that may influence economically important traits in goats [59,60]. Interestingly, *PPP1R3G*, a gene involved in glycogen metabolism and energy regulation, exhibited elevated densities of both SNPs and indels, suggesting increased functional variability in pathways related to metabolic regulation [61]. Overall, these findings further support the contribution of genomic variation to metabolic flexibility and environmental resilience in the Eghoria goat.

It should be noted, however, that the identification of genes with elevated SNP density should be interpreted with caution, as SNP density alone does not constitute definitive evidence of selection. Rather, it represents an exploratory measure that may highlight genomic regions of interest for further investigation. Confirmation of putative selection signatures will require complementary population genomic approaches, such as F_ST_-based analyses, haplotype-based methods, or other statistical tests specifically developed to detect natural or artificial selection across populations.

Among the validated candidate variants, several genes were associated with biological functions relevant to metabolism, physiological regulation, environmental adaptation, growth, and milk production traits, further supporting the functional relevance of the genomic variation observed in the Eghoria goat. Given the adaptation of this indigenous breed to extensive and semi-extensive production systems under challenging environmental conditions [4,9], variation in genes involved in metabolic efficiency, stress responses, and productive performance may contribute to its adaptive capacity and overall resilience. Notably, *SIRT1*, *GYS2*, *SI*, and *LIPC* are involved in metabolic regulation, energy homeostasis, glycogen metabolism, nutrient utilization, and lipid metabolism and have previously been associated with production and adaptive traits in livestock [62,63]. Similarly, *GYS2* plays a central role in glycogen metabolism and has been linked to metabolic adaptation in goats exposed to cold or high-altitude environments [64,65]. *SI* and *LIPC* are involved in carbohydrate digestion and lipid metabolism, respectively, and have been associated with efficient nutrient utilization and milk fat composition in goats [66]. Furthermore, calcium signaling genes, including *CACNA1C* and *CACNA2D1*, have previously been linked to growth-related traits in sheep [67]. Finally, *MYLK*, a gene involved in muscle contraction and cellular signaling, has been associated with body size and growth-related traits in goats [68]. Collectively, these findings suggest that variation in genes involved in nutrient metabolism, calcium signaling, and growth may contribute to the adaptive and productive characteristics of the Eghoria goat.

Importantly, validation of candidate missense SNPs using the MassARRAY platform confirmed the prioritized variants at the individual level, reinforcing the robustness of the whole-genome variant discovery and prioritization pipeline. The observed allele frequency distributions further indicate that several validated variants are maintained at appreciable frequencies within the studied population. Collectively, these findings highlight the value of integrating whole-genome sequencing with targeted validation for the genomic characterization of indigenous livestock populations.

Functional annotation of the validated missense variants was further supported by SIFT predictions and GERP conservation scores. Although most variants were predicted to be tolerated, three missense variants were classified as deleterious by SIFT, suggesting that these substitutions may have functional consequences and therefore warrant further investigation. The generally low or unavailable GERP scores indicate that evolutionary conservation alone should not be considered sufficient evidence for functional importance, highlighting the need for future experimental validation. Furthermore, the biological interpretation of variants in livestock species remains challenging due to the limited availability of species-specific in silico prediction tools. Specifically, PolyPhen-2 and CADD were not used because these tools are not fully supported for *Capra hircus* variant annotation. Furthermore, although PROVEAN represents an alternative tool for predicting the functional impact of amino acid substitutions, SIFT and GERP were selected because they are directly integrated into the Ensembl Variant Effect Predictor (VEP), providing a consistent annotation framework for the identified variants. Therefore, functional annotation was based on SIFT predictions together with GERP conservation scores as unlike human genomics, where multiple validated algorithms are available, several widely used tools are either unavailable or not validated for the *Capra hircus* genome. Consequently, integrating the available computational predictions with experimental validation is essential for improving confidence in the functional relevance of candidate variants.

The findings of the present study have important implications for the conservation and sustainable management of indigenous goat genetic resources in Greece. The identification of potential functionally relevant genomic variation associated with metabolism, immune response, signaling pathways, and environmental adaptation further highlights the biological value of this indigenous population. In the context of ongoing climate change and increasing environmental pressures, locally adapted livestock populations are considered particularly important reservoirs of adaptive genetic diversity [7,69]. Variants associated with metabolic flexibility, stress response, and physiological regulation may contribute to resilience under challenging production environments, including heat stress, limited nutritional resources, and disease-related pressures. Therefore, the genomic characterization of indigenous populations such as the Eghoria goat may support future strategies aimed at improving sustainability and resilience in small ruminant production systems.

Finally, several strengths and limitations of the present study should be considered. A significant strength lies in the integration of whole-genome variant discovery, functional enrichment analysis, and experimental validation within an indigenous and understudied goat population. Notably, the combination of pooled whole-genome sequencing (WGS) with individual-level MassARRAY validation offered a cost-effective and efficient methodology for identifying and confirming potentially functional variants. However, certain limitations should be acknowledged. A limitation of the present study is the use of pooled whole-genome sequencing during the variant discovery phase. Although it represents a cost-effective approach for genome-wide variant identification, it does not permit accurate estimation of individual allele frequencies, determination of individual genotypes, estimation of heterozygosity directly from the sequencing data, reconstruction of individual haplotypes, or comprehensive population genetic analyses. Furthermore, pooled sequencing has reduced sensitivity for detecting low-frequency variants, meaning that some rare, potentially adaptive alleles may have remained undetected. Nevertheless, the primary objective of this study was to identify potentially functional genomic variants rather than to characterize population structure or estimate genetic diversity. To strengthen the robustness of our findings, candidate variants identified through pooled sequencing were subsequently validated in an independent cohort of Eghoria goats using MassARRAY genotyping. This complementary approach combines the broad variant discovery capability of pooled sequencing with the accuracy of individual genotyping, providing greater confidence in the identified candidate variants.

Another limitation of the present study is the relatively small number of animals included in the whole-genome sequencing discovery phase. Although these animals were selected to represent the indigenous Eghoria goat population, the limited sample size may not fully capture the complete spectrum of genetic diversity within the breed, particularly low-frequency and rare variants. Furthermore, because variant discovery was based on pooled sequencing of a limited number of animals, the observed allele frequencies should be interpreted only as representative of the sampled pools and should not be generalized to the entire Eghoria goat population. Therefore, the detected polymorphisms should be regarded as candidate variants identified within the sampled population rather than an exhaustive catalogue of breed-specific variation. Future studies involving larger cohorts and individual whole-genome sequencing will provide a more comprehensive characterization of the genomic diversity of the Eghoria goat and facilitate the identification of additional functional variants associated with economically important traits.

Another limitation of the present study is the absence of genotype–phenotype association analyses. Consequently, although several candidate genes identified here have previously been associated with biologically relevant processes or economically important traits, the functional significance of the detected variants in the Eghoria goat population remains hypothetical. Therefore, the identified variants should be considered promising candidate markers rather than confirmed functional variants. Future studies integrating detailed phenotypic records, larger animal cohorts, and functional validation experiments will be essential to establish the biological relevance of these variants. Furthermore, although whole-genome sequencing enabled genome-wide variant discovery, the present study focused on coding variants because functional annotation and interpretation of non-coding regions remain comparatively limited in non-model livestock species, such as the goat. Nevertheless, regulatory variants located in promoters, enhancers, untranslated regions, and other non-coding elements are also likely to contribute to gene regulation, adaptation, and phenotypic diversity. As genomic resources improve, integrating coding and non-coding variation with transcriptomic and epigenomic data will provide a more comprehensive understanding of the molecular basis of economically important traits and environmental adaptation. Finally, comparative genomic analyses with other goat breeds were beyond the scope of the present study. Accordingly, it was not possible to distinguish Eghoria-specific polymorphisms from variants that are widespread across the species. Future comparative analyses including indigenous and commercial breeds will help identify breed-specific variants and further clarify the genetic basis of adaptation in the Eghoria goat.

## 5. Conclusions

In conclusion, the present study provides one of the first comprehensive whole-genome characterizations of the indigenous Greek Eghoria goat, combining variant discovery, functional annotation, and experimental validation. The identified coding variants expand the currently available genomic resources for this native breed and demonstrate the reliability of the applied whole-genome sequencing and validation strategy. These findings provide a valuable genomic foundation for the conservation, genetic improvement, and sustainable utilization of indigenous goat populations. Future studies integrating larger populations, detailed phenotypic records, and functional validation will facilitate the identification of genetic markers associated with adaptive and economically important traits, supporting the implementation of genomic-assisted breeding and conservation programs.

## Figures and Tables

**Table 1:** Top GO (BP, MF, CC) and KEGG pathways enrichment results for genes harboring prioritized missense SNPs in the Greek Eghoria Goat.

Top Enriched Biological Process Terms	Top Enriched Molecular Function Terms	Top Enriched Cellular Component Terms	Top Enriched KEGG Terms
Regulation of response to stimulus, Multicellular organism development, Anatomical structure development, Cell differentiation, Cellular developmental procedures, Developmental procedures, Multicellular organismal procedures, Negative regulation of biological procedures, Cellular component organization, Cellular response to stimulus	Monoatomic cation channel activity, Metal ion transmembrane transporter activity, Monoatomic ion channel activity, Channel activity, Passive transmembrane transporter activity, Phosphotransferase activity alcohol group as acceptor, Transporter activity, Enzyme binding, Catalytic activity acting on a protein, Catalytic activity	External encapsulating structure, Extracellular matrix, Plasma membrane region, Cell periphery, Plasma membrane, Nuclear lumen, Neoplasm, Membrane-enclosed lumen, Organelle lumen, Intracellular organelle lumen	ECM-receptor interaction, ABC transporters, Taste transduction, Complement and coagulation cascades, Lysine degradation, Arrhythmogenic right ventricular cardiomyopathy, Cytoskeleton in muscle cells, Calcium signaling pathway, Neuroactive ligand-receptor interaction, Metabolic pathways (Starch and sucrose metabolism)

**Table 2:** Top GO (BP, MF, CC) enrichment results for genes harboring exonic indels in the Greek Eghoria Goat.

Top Enriched Biological Process Terms	Top Enriched Molecular Function Terms	Top Enriched Cellular Component Terms
Circulatory system development, System development, Multicellular organism development, Negative regulation of cellular procedures, Cellular component organization, Cellular component organization or biogenesis, Negative regulation of biological procedures, Anatomical structure development, Developmental procedures, Multicellular organismal procedures	Calcium-dependent protein serine/threonine phosphatase activity, Calmodulin-dependent protein phosphatase activity, GTPase regulator activity Nucleoside-triphosphate regulator activity, Sequence-specific DNA binding, Sequence-specific double-stranded DNA binding, Double-stranded DNA binding, DNA-binding transcription factor activity RNA polymerase ii-specific, Transcription regulator activity, DNA binding	Histone methyltransferase complex, Methyltransferase complex, Adherens junction, Nuclear lumen, Nucleoplasm, Membrane-enclosed lumen, Organelle lumen, Intracellular organelle lumen, Cell periphery, Plasma membrane

## Data Availability

The datasets analyzed during the current study are available from the corresponding author upon reasonable request.

## Supplementary Materials

The following supporting information can be downloaded at: https://www.mdpi.com/article/10.3390/cimb48080778/s1.

## Abbreviations

The following abbreviations are used in this manuscript:

BP	Biological Process
CC	Cellular Component
GO	Gene Ontology
HWE	Hardy–Weinberg equilibrium
INDEL	insertions and deletions
KEGG	Kyoto Encyclopedia of Genes and Genomes
MF	Molecular Function
SNP	Single Nucleotide Polymorphism
WGS	Whole-Genome Sequencing
